# Neurological Disorders following COVID-19 Vaccination

**DOI:** 10.3390/vaccines11061114

**Published:** 2023-06-19

**Authors:** Ying Yang, Lisu Huang

**Affiliations:** Department of Infectious Diseases, Children’s Hospital, Zhejiang University School of Medicine, National Clinical Research Center for Child Health, Hangzhou 310052, China; 6510124@zju.edu.cn

**Keywords:** neurological disorder, COVID-19 vaccine, vascular factors, immune factors, infectious factors, functional factors

## Abstract

Nowadays, people all over the world have been receiving different types of coronavirus disease 2019 (COVID-19) vaccines. While their effectiveness has been well recognized, various post-vaccination disorders are not fully understood. In this review, we discuss neurological disorders related to vascular, immune, infectious, and functional factors following COVID-19 vaccination, and attempt to provide neuroscientists, psychiatrists, and vaccination staff with a reference for the diagnosis and treatment of these diseases. These disorders may present as a recurrence of previous neurological disorders or new-onset diseases. Their incidence rate, host and vaccine characteristics, clinical manifestations, treatment, and prognosis differ significantly. The pathogenesis of many of them remains unclear, and further studies are needed to provide more evidence. The incidence rate of severe neurological disorders is relatively low, most of which are reversible or treatable. Therefore, the benefits of vaccination outweigh the risk of COVID-19 infection, especially among fragile populations.

## 1. Introduction

**Figure 1:**
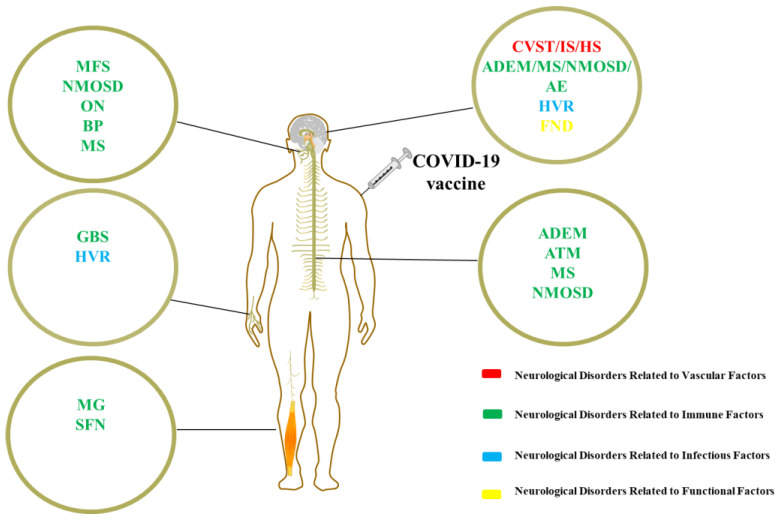
Neurological disorders following COVID-19 vaccination: CVST cranial venous sinus thrombosis; IS ischemic stroke; HS hemorrhagic stroke; ADEM acute disseminated encephalomyelitis; ATM acute transverse myelitis; MS multiple sclerosis; GBS Guillain–Barre syndrome; MFS Miller–Fisher syndrome; NMOSD neuromyelitis optica spectrum disorder; ON optic neuritis; AE autoimmune encephalitis; MG myasthenia gravis; SFN small fiber neuropathy; BP Bell’s palsy; HVR herpes virus reactivation; FND functional neurological disorder.

Coronavirus disease 2019 (COVID-19) is predominantly characterized by lung damage and hypoxia, leading to systemic complications and even death. As of the writing of this review, the World Health Organization has reported over 700 million confirmed cases, including over 6 million deaths [1]. COVID-19 vaccines have provided new hope for effectively combating the deadly pandemic. Several vaccines were developed during the pandemic, including inactivated or attenuated virus vaccines, recombinant protein vaccines, virus vector-based vaccines, and messenger RNA (mRNA) vaccines [2]. Safe vaccination with high uptake is crucial to the COVID-19 pandemic response. However, since the emergency authorization of different COVID-19 vaccines, there have been several reports of related diseases, with neurological complications being of significant concern, as they may be persistent and leave sequelae [3]. Almost all types of COVID-19 vaccines may cause neurological complications, and although the association between these vaccines and neurological complications is not yet clear, multiple hypotheses attempt to explain it. Many of these hypotheses are inferred from previous research about other vaccines [4]. Most cases of neurological disorders were anecdotal reports, and more in-depth analysis is needed. In this review, the authors divided them into four categories: neurological disorders related to vascular, immune, infectious, and functional factors, and summarized their characteristics (Figure 1). It aims to provide neuroscientists, psychiatrists, and vaccination staff with a reference for the diagnosis and treatment of these diseases.

## 2. Discussion

### 2.1. Neurological Disorders Related to Vascular Factors

Cerebrovascular disease is not uncommon among COVID-19 vaccine recipients [5]. They can range from mild transient cerebral ischemia to fatal cerebrovascular events. Recently, vascular events associated with vaccine-induced immune thrombotic thrombocytopenia (VITT) have drawn much attention. It is speculated that platelet factor-4 (PF4) /polyanion complexes play a pathogenic role. Specific ingredients in the vaccine trigger a strong anti-PF4 immune response, leading to thrombosis with thrombocytopenia syndrome [6]. VITT- and non-VITT-associated vascular events exhibit different clinical features.

#### 2.1.1. Cranial Venous Sinus Thrombosis

Cranial venous sinus thrombosis (CVST) is a cerebrovascular disease with blocked blood flow due to venous sinus thrombosis. It has been reported that over 70% of CVST cases following COVID-19 vaccination are related to VITT, which occurs in recipients of adenoviral vector-based vaccines [7,8]. Despite its low incidence (0.00009% per dose, 0.0005% per individual), it has a high mortality rate (47%) [9,10]. Most cases occur in individuals under 60 years old, with women being more commonly affected, and often accompanied by thrombosis in other parts of the body. The peak onset is between 4 days and 4 weeks after vaccination. Persistent headache is the most common clinical manifestation, along with sensory abnormalities, visual impairment, and so on [9]. Blood tests typically show decreased platelet count, increased D-dimer, and the presence of anti-PF4 antibodies. For suspected cases, cranial magnetic resonance imaging (MRI) combined with contrast-enhanced venous magnetic resonance angiography can aid in the diagnosis [11]. A high dose of intravenous immunoglobulin (IVIG) plus non-heparin anticoagulation is the main therapy strategy, while steroids may be another treatment option to improve the platelet level [6,12]. Nevertheless, CVST cases following COVID-19 vaccination outside the spectrum of VITT have also been reported [8]. Compared to VITT-CVST, non-VITT-CVST shows its own characteristics [7,13] (Table 1).

#### 2.1.2. Ischemic Stroke

Ischemic stroke (IS) is a cerebrovascular disease featured by brain infarction due to the occlusion of cerebral arteries. Its incidence among the COVID-19 vaccine recipients has been reported to range from 0.29 to 1.76/million vaccinations [14,15,16]. Cases of IS have been found after receiving various vaccine types [15]. Previous reports have reached different conclusions on whether vaccination increases the risk of IS. A study based on hospitalized patients recently vaccinated with mRNA COVID-19 vaccines showed that the incidence of IS was within the local baseline for the same period. They explained these cases by pre-existing cardiovascular risk factors rather than vaccines [15]. Another study monitored over 10 million vaccine-eligible members and found no increased IS risk after receiving the BNT162b2 vaccine [17]. A similar result was found in France among adults under 75 years old who received mRNA vaccines within 3 weeks [18]. However, data on approximately 30 million vaccinated patients in the United Kingdom showed that the risk of IS significantly increased after BNT162b2 vaccination [19]. IS after vaccination is characterized by advanced age (mean age of 68~71 years) and is predominantly male [14,15,20]. Many of these patients have previous high-risk factors for stroke. In an observational study based on hospitalized patients after mRNA vaccination, all patients had at least one cardiovascular risk factor (old age, hypertension, hyperlipidemia, diabetes, heart disease, obesity, smoking, atrial fibrillation, etc.) [14]. It is worth mentioning that atrial fibrillation is a risk factor only in female patients [21]. Diabetes is also a study-proven risk factor for IS, with the risk found to be higher in patients with diabetes [22]. The average interval from vaccination to symptoms is 9 days [14,15]. The macrovascular type is the most common subtype, and the anterior circulation is often involved [14,15,23]. Some patients receive revascularization therapy, including intravenous thrombolysis and mechanical thrombectomy, while others receive standardized care [14]. There was no difference in mortality rates between vaccinated IS patients and unvaccinated IS patients [23]. It is worth noting that some cases of IS are related to VITT, accounting for 3.1% [15]. In the VITT-IS group, female and younger patients are more common, many of whom are recipients of adenoviral vector-based vaccines and often accompanied by thrombosis in other sites [24]. Most patients lack previous cardiovascular risk factors, and intravenous thrombolysis is contraindicated [25,26]. High-dose IVIG should be initiated immediately, and non-heparin anticoagulant therapy should start 24 h after stroke onset. Mechanical thrombectomy is considered an effective treatment [27,28].VITT-IS is prone to conversion into malignant embolism (up to 41%) and has a high mortality rate (up to 20.8%) [25].

#### 2.1.3. Hemorrhagic Stroke

Hemorrhagic stroke (HS) is a cerebrovascular disease characterized by cerebral vascular rupture and bleeding. It is reported that the incidence of HS after COVID-19 is 364.7 events per million person-years [17]. HS has distinct gender differences, with a higher risk for females [29]. The occurrence of HS is related to race, with a high proportion in Asian populations [30]. HS occurs after various types of COVID-19 vaccines, especially mRNA vaccines [31]. The pathogenesis of the disease has not been fully exposed. Since most cases lack thrombocytopenia, they may not share a similar mechanism as VITT [17]. Whether COVID-19 vaccination increases the risk of HS remains controversial. A study found that after BNT162b2 vaccination, the risk of HS increased, and the incidence rate ratio (RR) was 1.24 (95% confidence interval (CI) 1.07~1.43) [29]. The report claimed that after being vaccinated with BNT162b2, there were 60 extra cases of HS per 10 million people in 1~28 days [29]. Additional studies further indicated that the risk of bleeding caused by BNT162b2 was specific to the brain rather than to other parts of the body [31]. However, in another study among BNT162b2 recipients, no association was found with HS, with a RR of 0.90 (95% CI 0.72~1.13) [17]. The mortality rate of HS is quite high, reaching 23% in a specific group of adults under 75 years old in France [18]. The mortality rate of COVID-19 in patients with previous HS has increased, making vaccination highly necessary [32]. However, the safety and effectiveness of the COVID-19 vaccine in this population remain unclear due to the lack of sufficient research.

### 2.2. Neurological Disorders Related to Immune Factors

There are various kinds of neurological disorders related to immune factors reported following COVID-19 vaccination. However, their pathogenesis is still under-researched, so scholars attempt to infer from other vaccines. The most popular hypothesis is molecular mimicry. Based on the similarity between pathogen-derived peptides in vaccines and the human nervous system, the immune response targeting the nervous system may be triggered. For example, mice immunized with influenza virus vaccines produced specific antibodies, supporting the association between influenza vaccines and Guillain–Barre syndrome [33]. COVID-19 vaccines could theoretically trigger cross-reactivity with brain tissue, spinal cord, nerve fibers, and neuromuscular junctions, inducing immune-related neurological disorders. The presence of various antibodies in patients with immune-related neurological disorders after the COVID-19 vaccines further confirms this hypothesis. However, a study conducted in the United Kingdom showed that there was no structural similarity between COVID-19 protein and peripheral nerve protein, although the possibility that the post-translational modification led to a similarity could not be ruled out [34]. The second hypothesis is bystander activation, which means adaptive immune cells are activated by cytokines induced by non-homologous antigens [35]. Susan van Aalst et alreported the bystander activation of irrelevant CD4 + T cells following vaccination occurs in the presence and absence of an adjuvant [36]. Bystander activation is sufficient to induce autoimmune disease, and it seems to better explain the short interval of some neurological disorders after COVID-19 vaccination [37]. In addition, vaccines activate innate immunity through pattern recognition receptors. The adjuvant of the COVID-19 vaccine has been reported to be able to activate toll-like receptors 3, 7, and 9 and produce interferon-α [38]. Interferon-α is related to Bell’s palsy after COVID-19 vaccination [39].

#### 2.2.1. Bell’s Palsy

Bell’s palsy (BP) is an acute peripheral facial nerve paralysis, accounting for 35.7% of all neurological disorders reported after COVID-19 vaccination [40]. The incidence has been estimated to range from 42.8 to 106 cases per 100,000 person-years [41,42]. It is most commonly reported to occur after mRNA vaccines but can also be seen with the inactivated or adenoviral vector-based COVID-19 vaccines [43]. BP occurs after the first or second dose of the vaccine, with a higher occurrence rate after the first dose [44]. There are reports of recurrence in patients with a previous history of BP [45]. The interval from vaccination to palsy is between 6 and 14 days [46,47]. The occurrence has gender differences: It is more common in females after mRNA vaccines but more common in males after inactivated vaccines [41,48,49]. Age difference has been found in some studies, with older people having a higher risk [45]. The mean age ranges from 49.7 to 62.6 years old [29,49,50]. It shows selection in location, with the left side being more commonly affected than the right side [46]. IVIG, steroids, and plasma exchange are first-line treatments, with most patients gaining recovery [44]. Studies draw different conclusions on the correlation between BP and the COVID-19 vaccines. A case-control study on 37 patients with facial nerve palsy who were recently vaccinated with the Pfizer-BioNTech vaccine failed to confirm the association between vaccines and BP (odds ratio (OR) 0.84, 95% CI, 0.37~1.90) [51]. However, a study conducted in Hong Kong assessed the risk of BP within 42 days following vaccination with BNT162b2 or CoronaVac and found a significantly increased risk (OR 2.385, 95% CI 1.415~4.022 for CoronaVac and OR 1.755, 95% CI 0.886~3.477 for BNT162b2) [41]. An analysis of a self-reporting database found BP was significantly more frequently reported after the administration of COVID-19 mRNA vaccines [50]. Fortunately, the excess risk of BP is low, with data from the largest healthcare provider in Israel showing approximately an extra 4.5 cases per 100,000 individuals among the highest associated group [45].

#### 2.2.2. Guillain–Barre Syndrome and Miller–Fisher Syndrome

Guillain–Barre syndrome (GBS) is an acute immune-mediated polyradiculoneuropathy that affects myelinated nerves. GBS is a severe complication after COVID-19 vaccination and a common cause of intensive care unit admission [2,52]. Most GBS cases were reported after the first dose of the vaccine [53,54,55,56]. The onset is usually within 2 weeks after vaccination, with a highly variable range from hours to weeks (within 6 weeks) [54,57,58]. The affected population’s age ranges widely, from 20 to 90 years old, and the incidence rate increases with age, with the mean age being in the 40 s to 60 s [54,55,57,59,60,61]. Most studies showed that males were the most susceptible gender [53,54,60]. Many studies have supported the high risk of GBS caused by adenoviral vector-based vaccines. A cohort study in the United States showed that the incidence rate of GBS during 1 to 21 days after Ad.26.COV2.S was 32.4/100,000 person-years, significantly higher than the background rate. In a head-to-head comparison, the adjusted RR of Ad.26.COV2.S for GBS was 20.56 [62]. Another study showed that within the 42-day window, the reporting rate of GBS after adenoviral vector-based vaccines was 5.57/100,000 person-years, while the background incidence rate was only 1.2~3.1/100,000 person-years [63]. Classic GBS is the most common clinical phenotype, with acute inflammatory demyelinating polyneuropathy (AIDP) as the most common neurophysiological subtype [59,60,64]. Anti-ganglioside antibodies are usually absent in these patients [60]. A recently reported GBS variant after the vaccine is highly characteristic: male dominance, frequent albuminocytological dissociation, and AIDP pattern [65]. Treatment usually includes IVIG and plasma exchange in severe patients, and the prognosis varies greatly [57]. Shaun Kai Kiat Chua et al conducted a review and pooled analysis of the data from 57 published cases. At the beginning of treatment, 60% reported improvement. However, post-treatment outcomes showed that 33% had a GBS disability scale score of 4 or more, indicating that they were either bedridden, needed ventilation support, or died [66]. A detailed customized rehabilitation program is necessary for them [67]. The recurrence risk of COVID-19 vaccination for patients with a GBS history is still uncertain, but some research results show the safety signal. A study showed that only 3.5% of patients have relapsed after vaccination [68]. Adája E Baars et al conducted a prospective, multicenter cohort study on patients with a history of GBS and found that none had a recurrence after vaccination [69].

Miller–Fisher syndrome (MFS) is rare after COVID-19 vaccination, characterized by ataxia, ametropia, ophthalmoplegia, and ametropia triad. It has been reported that the majority of MFS after the COVID-19 vaccine were males, and the median age was 65 years old. Various MFS phenotypes have been reported, including typical MFS, partial MFS, and even involvement of limbs and respiratory muscles. Cerebrospinal fluid (CSF) analysis shows typical albuminocytological dissociation and positive anti-GQ1b ganglioside antibody [70]. IVIG is the main treatment, and the prognosis for most patients is good [71,72]. Although it is a variant of GBS, it differs from GBS in many aspects [70,71,72] (Table 2).

#### 2.2.3. Myasthenia Gravis

Myasthenia gravis (MG) is an autoimmune disease caused by antibodies that bind to neuromuscular junction components, resulting in weakness of skeletal muscles. Cases of MG have been reported after COVID-19 vaccination, including both new-onset and worsening of previous MG [73,74]. These cases have been described following various COVID-19 vaccines and different doses [73,75,76]. The incidence reported varies greatly, ranging from 1% to 14.5% [77,78]. Acetylcholine receptor and muscle-specific receptor tyrosine kinase antibodies are only found in some patients [79]. New-onset patients usually have a short onset time (0~8 days) after vaccination, while the recurrence shows a longer interval (up to 3 months) [74,75]. Mild patients do not require additional medication, while severe ones need enhanced immunotherapy, such as steroids, IVIG, and plasma exchange. Patients with respiratory muscle involvement always receive tracheal intubation [78,80]. The prognosis may be variable. In a prospective study, patients only experienced temporary deterioration and responded well to treatment [77]. However, there were reports of fatal cases due to the MG crisis [81,82]. Patients with MG (pwMG) have poor outcomes of COVID-19 infection, which are also known as fragile populations. A retrospective observational study of MG patients in Brazil found that 87% of MG-COVID cases required intensive care, 73% needed mechanical ventilation, and 30% died [83]. COVID-19 infection in vaccinated MG patients would be milder, so this population needs vaccination during the epidemic. Some studies have shown the safety of the COVID-19 vaccine in pwMG. A study compared the deterioration rate of MG after COVID-19 vaccination with that of previous years and found that vaccination did not increase the risk of MG worsening [73]. The relationship between the stability of the previous MG and the deterioration after vaccination is still unclear. Some patients with unstable MG did not experience worsening symptoms, while others with well-controlled conditions deteriorated after vaccination [75].

#### 2.2.4. Demyelinating Diseases

##### Optic Neuritis

Optic neuritis (ON) is an inflammatory disorder that affects the optic nerve, with demyelination being a common pathology subtype. ON is relatively common after COVID-19 vaccination. Cases of ON have been reported following various vaccines, including inactivated vaccines, adenoviral vector-based vaccines, and mRNA vaccines [84,85,86]. Both the first and second doses of vaccine-related ON have been reported, with an interval time of 0~19 days [85,87,88]. One or both sides of the optic nerve will be affected, typically presenting with visual impairment and eye pain, rarely presenting only papilledema [87,88]. Myelin oligodendrocyte glycoprotein (MOG) IgG is present in some patients [85]. Patients usually receive steroid therapy, and severe ones need a combination of plasma exchange [89]. A study involving 715 patients found that affected individuals were mainly females, elderly, and American and Europe residents. A total of 73% of cases belonged to severe cases, and only 11% of patients completely recovered after treatments, although there were no fatal cases in this study. Since partial patients may ultimately be diagnosed with multiple sclerosis or neuromyelitis optica spectrum disorder, it is difficult to accurately determine the incidence rate from the reports [90]. 

##### Acute Transverse Myelitis

Acute transverse myelitis (ATM) is an acquired demyelinating disease of the spinal cord. ATM following COVID-19 vaccination is rare, with an incidence of approximately 1~4 cases per million people [61]. Interestingly, there were quite a few reports of a subtype called longitudinally extended transfer myelitis, which involves three or more vertebral segments [91]. The mean age of patients was typically in the 40 s to 50 s, and a large proportion of patients had previous autoimmune diseases [92,93]. Compared with COVID-19-related ATMs, vaccine-related ones show shorter intervals and milder clinical manifestations [92,93]. They occur after various types of COVID-19 vaccines, with the adenoviral vector-based vaccine being the most common [91,94,95,96]. Cases are characterized by quadriplegia or lower limb paralysis, transverse sensory level, and rectal or bladder dysfunction. The thoracic spine is the most frequently involved [92]. Some patients’ CSF may show an increase in leukocyte count and protein. Specific oligoclonal bands may appear in CSF, and MOG and aquaporin protein-4 (AQP4) antibodies are usually absent. MRI may show a high signal of the spinal cord on T2 or fluid-attenuated inversion recovery (FLAIR) sequence, and the lesion may involve an extensive spinal cord. High-dose steroids are the basic treatment, and in severe cases, a combination of IVIG, immunosuppressive agents, or plasma exchange is needed [97]. Most patients have a good prognosis, with only a few deaths [92]. Older age, the second dose of vaccine, and a modified Rankin score of ≥3 are indicators of poor prognosis [92].

##### Acute Disseminated Encephalomyelitis

Acute disseminated encephalomyelitis (ADEM) is an acute or subacute demyelinating disease that affects multiple parts of the central nervous system, accompanied by encephalopathy. According to reports, the incidence of ADEM following COVID-19 vaccination was as low as 0.36 cases per million people [98]. Unlike previous ADEM, ADEM after COVID-19 vaccination predominantly affects female adults, and most patients have no underlying diseases or immunosuppressive medication usage [99,100]. ADEM can occur among different vaccine recipients, with adenovirus vector-based vaccines dominating [100,101]. Most patients initially show clinical symptoms within 1 to 35 days after COVID-19 vaccination, which is shorter than other demyelinating diseases [100,102]. It may manifest as the only clinical condition or a part of multiple autoimmune syndromes [103]. MOG antibodies are present in the serum of some patients with COVID-19 vaccine-related ADEM [102]. The key therapy for severe patients includes IVIG, steroids, and plasma exchange [100]. ADEM usually presents as a monophasic course, although a few recurrent cases have been reported [104]. Fortunately, most patients have a good prognosis after treatment, with a few fatal cases [102]. 

##### Multiple Sclerosis

Multiple sclerosis (MS) is a central nervous system chronic immune-inflammatory demyelinating disease that can lead to irreversible disability. Patients with MS (pwMS) are at greater risk and have poorer outcomes of COVID-19 infection [105]. PwMS are considered fragile populations, and statements have been released in many countries that pwMS need vaccination against COVID-19 [106,107]. There are reports of new-onset and recurrent MS cases after COVID-19 vaccination [93,108]. According to a systematic review, the affected population was mainly young adults, with a mean age of 33.5 years [105]. Similar to the previously reported MS, the cases after COVID-19 vaccination are mainly females [93,109,110,111,112]. Studies showed that the mean onset time was 6 to 9 days, and sensory abnormality was the most common clinical manifestation [93,109]. MRI shows new emerging signals on the T2 and FLAIR sequences. The brain is the most commonly affected, followed by the spinal cord. Specific oligoclonal bands could be present in CSF [93]. MS usually occurs after adenoviral vector-based and mRNA vaccines [109,113]. It is worth mentioning that the MRI of some newly diagnosed cases showed a mix of new and old lesions, so we cannot completely rule out a previously pre-clinical MS [114]. Karlo Toljan et al. reported that some patients responded to high-dose steroids, while others needed additional plasmapheresis [114]. The lesions disappear in most patients after treatments, but unfortunately, a few could not recover to baseline [109]. Different conclusions have been drawn from studies about whether the COVID-19 vaccines increased the recurrence risk of MS. Epidemiological survey showed that after the first and second doses of vaccination, the acute recurrence rates were 0.65 to 2.1% and 0.85 to 1.6%, respectively. It is hard to separate these from natural recurrence rates since they were too close to non-vaccination ones during the same period [115,116]. However, other studies showed that COVID-19 vaccination increased the recurrence risk of MS due to different vaccine types and follow-up periods [113].

##### Neuromyelitis Optica Spectrum Disorder

Neuromyelitis optica spectrum disorder (NMOSD) is a neuro-inflammatory demyelinating disease in the optic nerves and spinal cord. Vaccines can be a trigger for NMOSD, including the COVID-19 vaccine. NMOSD can occur in various COVID-19 vaccine recipients, with adenoviral vector-based vaccines being the most common [100,117]. Some patients have previous autoimmune diseases [118]. Most patients reported the initiation of symptoms after receiving the first dose of the vaccine with a median interval of 6 to 10 days [100,117]. Females are more commonly affected, and the age ranges widely, from young people to the elderly [100,117,119]. Acute medullary syndrome is the most common clinical manifestation [100]. CSF analysis shows elevated monocyte lymphocyte and protein levels [117]. Most patients improve through high-dose steroids or plasma exchange, while some patients need maintenance treatments, including rituximab and azathioprine [117]. Even after active treatment, some patients may have sequelae, while elderly patients may experience death [100,119]. The specific biomarker of NMOSD (AQP4 antibody) is present in the majority of patients (up to 83.3%) [117]. Regarding clinical relapse after vaccination among patients with NMOSD, it has been reported that the mean occurrence time was 49.75 days with an incidence rate of 4.67%. Patients are mainly females, and AQP4 IgG or MOG IgG is usually present. Some patients need immunotherapy, such as rituximab, azathioprine, and steroids. The clinical recovery of most patients is well [117]. Recurrence seems to be related to the disease status before vaccination. A recent study revealed a higher risk of vaccination-associated relapse in untreated patients [120].

#### 2.2.5. Autoimmune Encephalitis

Autoimmune encephalitis (AE) is a disease where antibodies target brain tissue, leading to cognitive disorders, mental disorders, and seizures. AE after COVID-19 vaccination is rare. A systematic review showed 14 cases of AE after COVID-19 vaccination and summarized their clinical characteristics: It often occurs in young adults, with a median age of 32 [121]. Different from the COVID-19-related AE, the majority of AE after vaccination are females [121]. AE occurs after both adenoviral vector-based and mRNA vaccines, and most happen at the first dose [99]. Symptoms appear quickly after vaccination, with a median time interval of 5 days [121]. The N-methyl-D-aspartate-receptor (NMDAR) antibody and leucine-rich glioma inactivated gene-1 antibody are found only in some of the patients [99,122]. Consciousness decline and speech or memory disorder are common clinical manifestations [121]. Brain MRI suggests abnormal lesions on the cerebral cortex and deep gray matter, with the temporal lobe being the more commonly affected site [121]. Many patients may have MRI-negative findings, and a positron emission tomography/computed tomography is needed [121]. Inflammatory biomarkers of peripheral blood do not increase, but lymphocytes, protein, and glucose elevate in CSF [121,123,124]. Electroencephalography indicates θ and δ rhythm [121]. Patients show improvements through single or combined immunotherapy, including steroids, IVIG, and rituximab. However, sequelae are found among many patients [121]. Concern has been raised about the recurrence among patients with AE (pwAE) after the COVID-19 vaccination. A cross-sectional survey showed that only 3.3% of 121 pwAE recurred after inactivated COVID-19 vaccination, one recurred within 1 month, and three recurred 3 months later [125]. Unlike new-onset AE, the recurrent cases are mostly NMDARpositive. In addition, the median interval is significantly longer than that of new-onset AE.

#### 2.2.6. Small Fiber Neuropathy

Small fiber neuropathy (SFN) is a peripheral neuropathy involving small nerve fibers. Previous studies have shown that a large proportion of SFN cases occurring after the COVID-19 vaccine involve autoantibodies, indicating that the disease has the basis of autoimmunity [126,127,128]. One study found that the titers of antibodies to adrenergic receptors, muscarinic cholinergic receptors, and angiotensin-converting enzyme 2 increased in an SFN patient after the COVID-19 vaccine [127]. Antibodies to fibroblast growth factor receptor 3 were found in another case [126]. They may either be a new-onset case or a recurrence of the previous condition [129]. Acute sensory abnormality is one of the outstanding clinical features, such as burning dysesthesias, numbness, tingling sensation, and tinnitus [127,130]. Another feature is dysautonomia, and postnatal orthostatic tachycardia syndrome has been reported as a typical manifestation [127]. Electromyography shows normal peripheral nerves, while skin biopsy indicates that the density of nerve fibers decreases [126,130]. Patients with mild symptoms only need painkillers to release. Considering the autoimmune basis of the disease, the use of steroids and IVIG is also helpful [126,127]. Some patients recover quickly, while others experience symptoms for a prolonged period [126].

### 2.3. Neurological Disorders Related to Infectious Factors

Sporadic reports emerge about the occurrence of infectious diseases in the nervous system after COVID-19 vaccination. Although the precise mechanism is unknown, innate or cell-mediated immune defense failure caused by vaccines has been considered a potential factor [131]. A study by Walsh et al on the safety and immunogenicity of the BNT162b2 vaccine found a brief decrease in lymphocyte count after vaccination [132].

Herpes virus reactivation (HVR) refers to the proliferation of the herpes virus latent in the human body previously, leading to the worsening of infection. HVR after the COVID-19 vaccination, especially the varicella-zoster virus (VZV) causes great concern. Psichogiou et al claimed that temporary disability of VZV-specific CD8 + T cell function could reactivate the virus in the body [133]. The incidence was reported to be as high as 0.22%, significantly higher than that of non-vaccinated people [134]. Almost all types of COVID-19 vaccines may cause reactivation, especially the mRNA vaccines [135,136,137,138]. A study showed that mRNA COVID-19 vaccines were associated with higher reports of VZV reactivation compared with the influenza vaccine (OR 1.9, 95% CI 1.8~2.1) [139]. Mild cases present VZV radiculitis and neurofibroitis, while severe cases endanger the eyes and central nervous system [139]. Either the first or second dose of the vaccine faces this risk. A systematic review showed that there were more cases reported after the first vaccination [136]. A large portion of affected individuals previously have varying degrees of immunosuppression. A study found that up to 23% of patients were receiving immunosuppressive therapy or having autoimmune diseases [136]. Another risk factor is age, and the affected individuals have characteristics of advanced age. Compared to the elderly, the risk of VZV reactivation was reduced in people under 40 years old (OR 0.39, 95% CI 0.36~0.41) [139]. Other high-risk factors include diabetes and chronic hepatitis B virus infection [140]. No significant difference in the incidence between males and females was found [141]. The onset time depends on the type of recurrence: For those, the central nervous system is not involved, the mean onset time is 7.2 days, while for those where the central nervous system is affected, the mean time lengthens to 17.5 days [139]. A self-controlled case series and nested case-control study found that the high-risk period for VZV recurrence was from the first dose to 14 days after the second dose of the mRNA vaccines [137]. Most patients receive standard antiviral treatment, while some need hospitalization [139,141].

### 2.4. Neurological Disorders Related to Functional Factors

Neurological disorders related to functional factors following the COVID-19 vaccine are neither related to vaccine composition nor immunological factors [142]. They are contributing to biological, environmental, or psychosocial factors: misunderstanding of COVID-19, excessive stress associated with the pandemic, and the painful sensation of vaccination [143]. Among them, functional neurological disorder (FND) has drawn much attention.

FND is a disease related to brain network function, rather than the structure of the nervous system [144]. FND after vaccination has been reported with other vaccines, and COVID-19 vaccine-related FND has aroused great interest from clinicians due to it being widespread [145]. COVID-19 vaccine-related FND accounts for 2.88 to 3.5% of COVID-19 vaccine-related neurological complications [40,146]. Some patients with existing FND reported worsening symptoms, while new-onset cases are rapidly increasing. FND occurs after almost every type of COVID-19 vaccine [147]. There are diverse clinical manifestations, including motor, sensory, visual, and hypersensitivity [146]. The onset time varies from several minutes to weeks, but most cases attack suddenly with a mean onset time of 1 day [146,147]. Even with the intervention of psychologists, some patients remain with disabilities [148]. Compared with the COVID-19 related-FND, vaccine-related FND has unique characteristics. A comparing study found that patients were younger, female-dominated, had more acute onset, and had no neuropsychiatric abnormalities previously [148]. FND is treatable and requires early intervention to prevent chronic disease progression. A detailed explanation of the side effects of vaccines is an important measure for prevention [149].

## 3. Conclusions

A variety of neurological disorders may occur among individuals who have recently received the COVID-19 vaccines. These disorders can be classified into four categories: those related to vascular factors, immune factors, infectious factors, and functional factors, and some may be related to multiple factors. Their possible pathogenesis, incidence rate, host and vaccine characteristics, clinical manifestations, treatments, and prognosis differ significantly. Neurological disorders can present as new-onset cases or as a recurrence of existing diseases. The pathogenesis of many neurological disorders following the COVID-19 vaccines remains unclear, and more in-depth studies are needed to clarify current hypotheses and provide additional evidence. The incidence rate of severe neurological disorders is relatively low, and the benefits of vaccination outweigh the risk of COVID-19 infection, especially among fragile populations.

## Figures and Tables

**Table 1:** Clinical characteristics of VITT-CVST and non-VITT-CVST following COVID-19 vaccination.

	VITT-CVST	Non-VITT-CVST
Age	Younger	Older
Vaccine type	Adenoviral vector-based vaccines	Non-vector-based vaccines
Onset time	Shorter interval	Longer interval
Thrombocytopenia	Yes	Usually no
PF4 antibodies assay	Usually positive	Negative
D-dimer	Higher than 2000 μg/L	Lower than 2000 μg/L
Other risk factors for thromboses	Usually absent	Usually present
Intracerebral hemorrhage	More common	Less common
Thromboses in other sites	More common	Less common
Mortality	Higher	Lower
Treatment	Non-heparin anticoagulants and IVIG	Heparin anticoagulants

**Table 2:** Clinical characteristics of Guillain–Barre syndrome and Miller–Fisher syndrome following COVID-19 vaccination.

	Guillain–Barre Syndrome	Miller–Fisher Syndrome
Vaccine type	Most adenoviral vector-based vaccines	Various kinds of vaccines
Anti-ganglioside antibodies	Usually absent	Usually present
Respiratory muscle involvement	More frequent	Less frequent
Treatment	IVIG, plasma exchange	Usually IVIG only
Prognosis	Various	Good

## Data Availability

Not applicable.
